# Comparative In Vitro Evaluation of Tube Resistance in Glaucoma Drainage Devices with Intraluminal Polypropylene Threads

**DOI:** 10.3390/bioengineering13080923

**Published:** 2026-08-14

**Authors:** Arief Abdurrazaq Dharma, Sachiko Kaidzu, Andi Masdipa, Virna Dwi Oktariana Asrory, Masaki Tanito

**Affiliations:** 1 Department of Ophthalmology, Shimane University Faculty of Medicine, Izumo 693-8501, Shimane, Japan; 2 Faculty of Medicine, Universitas Muslim Indonesia, Makassar 90231, Indonesia; 3 Faculty of Medicine, Universitas Indonesia, Jakarta 10430, Indonesia; 4 Cipto Mangunkusumo Hospital, Jakarta 10430, Indonesia

**Keywords:** glaucoma drainage devices, Ahmed ClearPath, Paul Glaucoma Implant, Virna Glaucoma Implant, tube resistance

## Abstract

**(1) Background:** To compare tube resistance among glaucoma drainage devices (GDDs) with different tube geometries under various intraluminal polypropylene (PP) thread conditions and assess agreement between measured and formula-derived pressure–flow profiles. **(2) Methods:** Four GDDs were evaluated: Ahmed ClearPath Small Tube (ACP-ST), Paul Glaucoma Implant (PGI), Ahmed ClearPath (ACP), and Virna Glaucoma Implant (VGI) (n = 4/device). ACP-ST and PGI were tested under no-thread, 6-0 PP Corza, and 6-0 PP Ethicon conditions, whereas ACP and VGI were tested under no-thread, 4-0 PP Corza, and 3-0 PP Ethicon conditions. Saline was infused at 1.0–5.0 µL/min, and pressure was recorded using a transducer system. Tube and thread dimensions were measured using ImageJ and used to calculate formula-derived pressure from fluid dynamic equations. Comparisons used linear mixed-effects regression. **(3) Results:** In ACP-ST and PGI, both 6-0 PP threads significantly increased pressure compared with the no-thread condition, with no significant thread manufacturer-related differences. In ACP and VGI, all threaded conditions significantly increased pressure, and the 3-0 PP Ethicon condition produced higher pressure than the 4-0 PP Corza condition. Under threaded conditions, measured and formula-derived pressure values did not differ significantly in ACP-ST but differed significantly in PGI, ACP, and VGI. **(4) Conclusions:** Intraluminal PP threads increased GDD tube resistance. Manufacturer-related differences had a limited influence on resistance in small-lumen devices. Agreement between measured and formula-derived pressure varied among devices, highlighting the importance of experimental validation. These findings may help guide intraluminal thread selection and flow-restriction strategies during GDD implantation.

## 1. Introduction

Glaucoma drainage devices (GDDs) are widely used in the management of refractory glaucoma to provide an alternative pathway for aqueous humor outflow and reduce intraocular pressure (IOP) [1,2]. Although these devices are effective in lowering IOP, early postoperative pressure control remains a critical challenge [3,4,5,6]. Excessive aqueous outflow can result in hypotony and associated complications, such as shallow anterior chamber or choroidal detachment, whereas insufficient flow restriction may lead to suboptimal IOP reduction [1,7]. These early postoperative outcomes are largely governed by the hydraulic resistance within the drainage tube, which plays a central role in regulating fluid dynamics before stable bleb formation is established.

From a mechanical perspective, the tube segment of a GDD acts as a primary resistor that determines the initial pressure–flow relationship. Variations in tube diameter, length, and internal geometry can significantly influence resistance and, consequently, fluid outflow. While plate design and bleb characteristics contribute to long-term outcomes, tube resistance is particularly relevant in the early postoperative phase, where flow is not yet modulated by fibrous encapsulation [8,9,10]. Accordingly, the present study focuses specifically on the resistance characteristics of the tube component under controlled conditions.

Established GDDs, including Ahmed Glaucoma Valve and Baerveldt Glaucoma Implant, have long been used in clinical practice for the surgical management of refractory glaucoma [11,12,13,14,15]. Their clinical outcomes and flow-related characteristics have been investigated in previous studies [16,17]. Despite the clinical relevance of tube resistance, standardized comparative evaluations across newer GDD designs remain limited. Newer devices with different tube geometries have recently become available, including Ahmed ClearPath (ACP) (New World Medical Inc, Rancho Cucamonga, CA, USA), Ahmed ClearPath Small Tube (ACP-ST) (New World Medical Inc), Paul Glaucoma Implant (PGI) (Advanced Ophthalmic Innovations Pte Ltd., Singapore), and Virna Glaucoma Implant GL-51 (VGI) (PT Rohto Laboratories Indonesia, Bandung Barat, West Java, Indonesia). These four devices were selected because their tube resistance characteristics have not previously been evaluated and directly compared using a standardized experimental setup. In particular, the ACP-ST and PGI are recently introduced small-lumen GDDs that have attracted attention as potentially less invasive tube-shunt options. Their smaller tube geometry may allow a smaller entry tract while providing greater intrinsic hydraulic resistance, potentially reducing excessive aqueous outflow and the risk of early postoperative hypotony. These devices differ in tube diameter, lumen design, and intended resistance profile. The ACP represents a non-valved implant with a conventional tube design, whereas the PGI and ACP-ST incorporate smaller-lumen concepts intended to increase intrinsic flow resistance [16,18,19,20,21,22]. The VGI is a newer, low-cost, non-valved GDD developed for aqueous humor drainage in Indonesia [23,24]. Therefore, direct comparison of these devices under standardized experimental conditions is necessary to clarify their intrinsic tube resistance.

To further modulate aqueous outflow, intraluminal occlusion techniques using polypropylene (PP) threads are commonly employed in clinical practice [16,25]. These threads partially occupy the tube lumen, thereby increasing resistance in a controllable manner and reducing the risk of early postoperative hypotony. The extent of resistance depends on both thread size and the geometry of the tube, suggesting that the interaction between device design and thread insertion may vary across different GDDs. However, this relationship has not been consistently quantified, and comparative data across newer devices remain scarce.

In the present study, the selection of thread conditions was based on both device configuration and thread characteristics. ACP-ST and ACP are supplied with preloaded intraluminal PP threads by the manufacturer, whereas PGI and VGI require manual thread insertion. In small-lumen devices, such as ACP-ST and PGI, 6-0 PP threads are clinically relevant because ACP-ST is supplied with a preloaded 6-0 PP Corza thread. Comparing 6-0 PP threads from different manufacturers also allows assessment of whether manufacturer-related differences affect tube resistance when the United States Pharmacopeia (USP) thread size is the same. This is of relevance given that polypropylene threads are classified according to the USP sizing system, which delineates allowable ranges of diameter and tensile strength rather than precise dimensions. Consequently, threads with the same nominal USP size may exhibit slight variations in measured diameter between manufacturers. In larger-lumen devices, such as ACP and VGI, these differences may alter the proportion of the lumen occupied by the thread and thereby influence flow resistance.

From a fluid mechanics standpoint, resistance is primarily determined by lumen geometry and flow conditions. Formula-derived pressure estimation was used as a mechanistic reference to determine whether measured tube resistance could be explained by tube and thread geometry alone. However, measured flow behavior may deviate from formula-derived values because of non-circular lumen geometry, manufacturing variability, eccentric thread positioning, and interactions between the tube wall and intraluminal thread. Comparing measured and formula-derived pressure–flow profiles may help identify whether device-specific deviations arise from ideal geometric assumptions or non-ideal intraluminal flow behavior.

Accordingly, the objective of this study was to quantify and compare tube resistance among ACP-ST, PGI, ACP, and VGI under standardized in vitro conditions. Specifically, we evaluated whether resistance differed between 6-0 PP threads from different manufacturers in small-lumen devices and whether measured pressure–flow profiles agreed with formula-derived pressure estimates based on tube and thread geometry.

## 2. Materials and Methods

### 2.1. Materials

**Figure 1:**
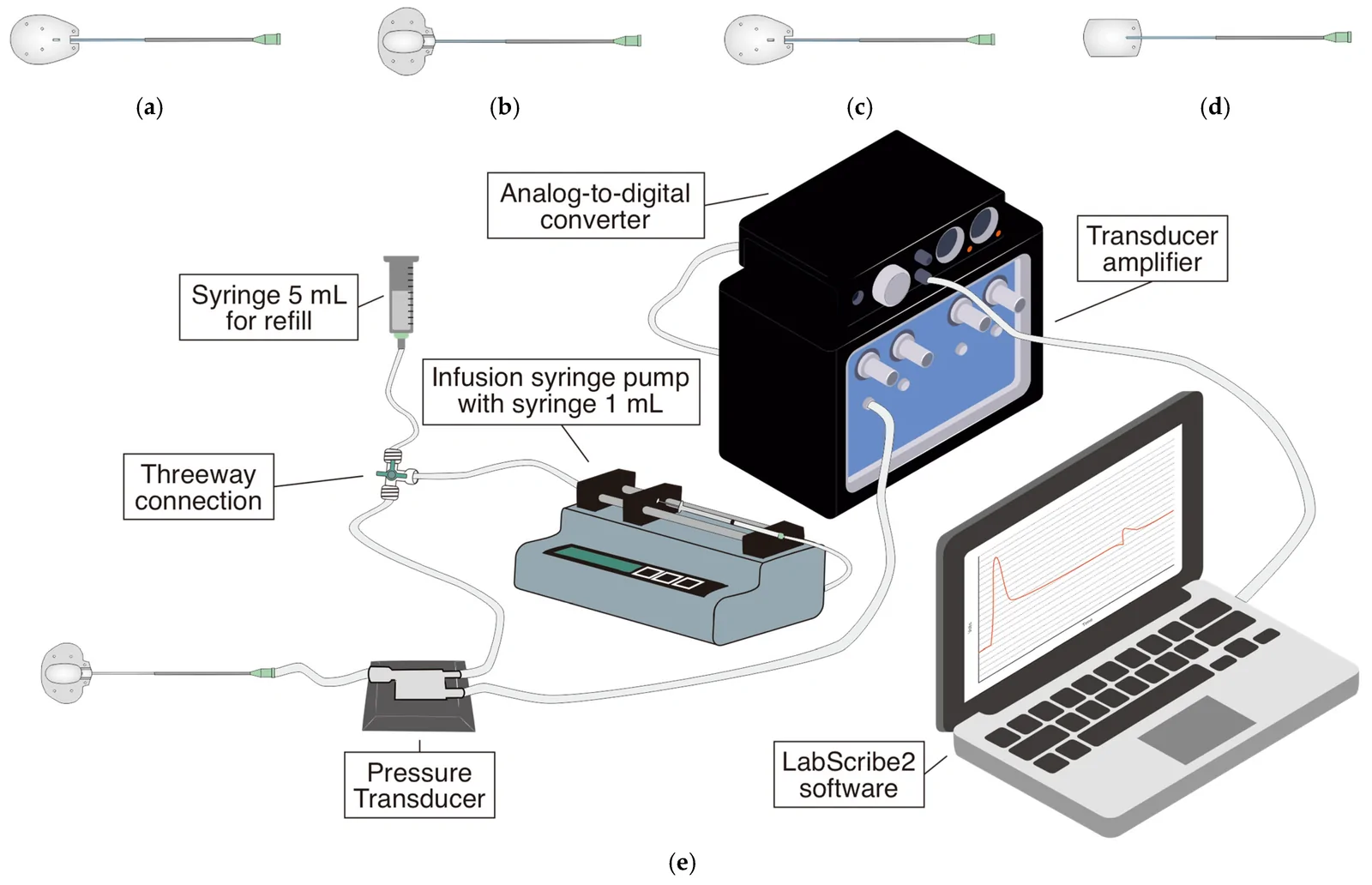
Experimental setup for in vitro pressure resistance measurement. (**a**) Ahmed ClearPath–Small Tube (ACP-ST); (**b**) Paul Glaucoma Implant (PGI); (**c**) Ahmed ClearPath (ACP); (**d**) Virna Glaucoma Implant (VGI); (**e**) schematic overview of the experimental setup.

The present study evaluated four types of GDDs: the ACP-ST (Figure 1a), the PGI (Figure 1b), the ACP (Figure 1c), and the VGI (Figure 1d). According to manufacturer-reported specifications (by person communication and/or catalogue specification), the tube inner and outer diameters were 0.13 and 0.46 mm for ACP-ST, 0.13 and 0.46 mm for PGI, 0.31 and 0.64 mm for ACP, and 0.33 and 0.64 mm for VGI, respectively. The corresponding plate surface areas were 250 mm^2^, 342.1 mm^2^, 250 mm^2^, and 200 mm^2^, respectively. For each device, four independent samples were analyzed (n = 4 per group), and each measurement was obtained from a distinct device without repeated measurements.

For ACP-ST and PGI, three experimental conditions were tested: the absence of an intraluminal thread, the presence of a 6-0 PP thread (Ethicon Inc., Raritan, NJ, USA), and the presence of a 6-0 PP thread (Corza Medical, Waltham, MA, USA). ACP-ST is supplied with a preloaded 6-0 PP thread, which, according to information provided by New World Medical, is manufactured by Corza Medical. For ACP and VGI, three conditions were also evaluated: the absence of an intraluminal thread, the presence of a 3-0 PP thread (Ethicon Inc., Raritan, NJ, USA), and the presence of a 4-0 PP thread (Corza Medical, Waltham, MA, USA). Similarly, ACP is supplied with a preloaded 4-0 PP thread, which, according to information provided by New World Medical, is manufactured by Corza Medical. Thread sizes were selected based on their clinical use and compatibility with the lumen size of each device. A 6-0 PP thread was tested in the small-lumen tubes of the ACP-ST and PGI, whereas both 3-0 and 4-0 PP threads were tested in the larger-lumen tubes of the ACP and VGI, reflecting clinically used device–thread combinations.

### 2.2. “Measured Pressure” Measurements

The in vitro pressure resistance measurements were conducted using a fluidic system that had been adapted from previously established methods [16], as illustrated in Figure 1e. Physiological saline was infused using a 1 mL syringe (Terumo Corporation, Tokyo, Japan) connected to an infusion syringe pump (SP101i; KD Scientific, Holliston, MA, USA) to generate controlled flow rates. A 5 mL syringe manufactured by Terumo Corporation was connected via a three-way connector and used as a refill reservoir during the experiment. The saline solution was hypothesized to possess physical properties analogous to water at ambient temperature, with a dynamic viscosity ranging from 0.89 to 1.00 mPa·s. All measurements were conducted at a controlled ambient temperature of 25 °C.

The infusion system was connected via infusion tubing (JMS Co., Ltd., Tokyo, Japan) and a three-way connector to a pressure transducer (BLPR2; World Precision Instruments, Sarasota, FL, USA). The transducer was connected to a transducer amplifier (SYS TBM4M; World Precision Instruments) and an analog-to-digital converter (LAB-TRAX-4/16; World Precision Instruments). Pressure data were continuously recorded and analyzed using LabScribe2, version 2.42500 software (World Precision Instruments). Each glaucoma drainage device (ACP-ST, PGI, ACP, and VGI) was connected to the system using appropriately sized needles (Terumo Corporation, Tokyo, Japan) to ensure a stable interface. For ACP-ST and PGI, a 21G 1/2-inch needle was utilized, whereas for ACP and VGI, a 19G 1/2-inch needle was employed. To prevent leakage at the point of connection between the needle and the GDD tube, adhesive glue (TOMBOW Pencil Co., Ltd., Aichi, Japan) was applied at the interface. A visual inspection of the system was conducted to ascertain the absence of leakage at the connection interface.

The intraluminal threads were inserted along the full length of the tube lumen under each threaded condition prior to measurement. GDD tubes were measured at their original, uncut lengths. The measured tube lengths (mean ± SD, n = 4) were 31.1 ± 0.2 mm for ACP-ST, 30.1 ± 0.4 mm for PGI, 32.5 ± 0.3 mm for ACP, and 33.7 ± 0.5 mm for VGI. Prior to conducting the measurements, the pressure transducer was calibrated to a zero baseline. Subsequently, physiological saline was infused at flow rates of 1.0, 1.5, 2.0, 2.5, 3.0, 4.0, and 5.0 µL/min, and the corresponding pressure values were meticulously recorded.

### 2.3. “Formula-Derived Pressure” Calculations

**Figure 2:**
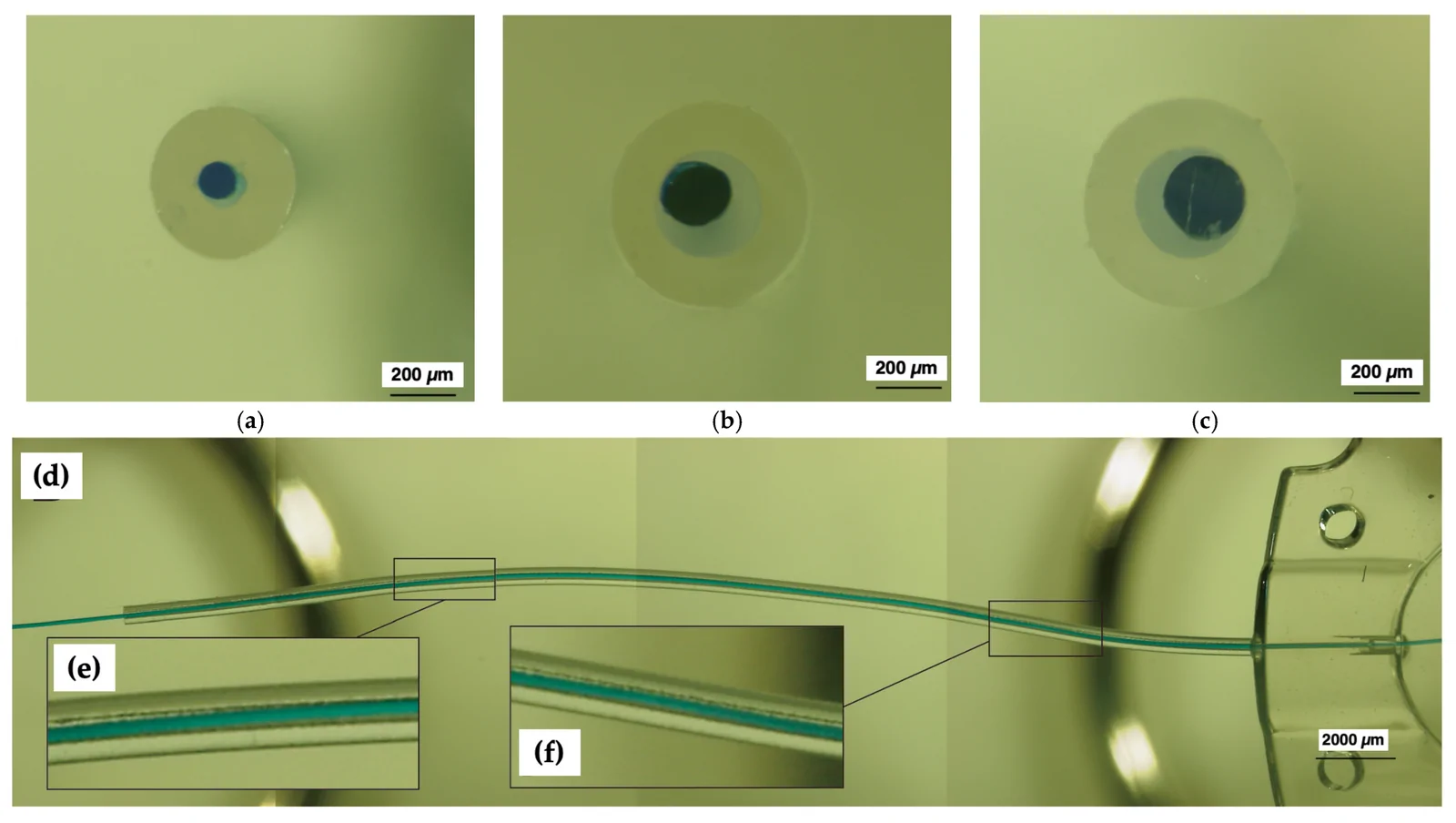
Microscopic images of glaucoma drainage device tubes with intraluminal polypropylene (PP) threads. (**a**) Ahmed ClearPath–Small Tube (ACP-ST) with 6-0 PP thread; (**b**) Ahmed ClearPath (ACP) with 4-0 PP thread; (**c**) Virna Glaucoma Implant (VGI) with 3-0 PP thread; (**d**) Paul Glaucoma Implant (PGI) with a 6-0 polypropylene thread, showing the longitudinal course of the thread within the tube. (**e**,**f**) Enlarged views of selected regions in panel (**d**), illustrating local variation in thread position within the tube lumen. Scale bars: 200 µm in (**a**–**c**) and 2000 µm in (**d**).

The formula-derived pressure values were calculated using geometric measurements obtained from microscopic images of each device and thread condition. Microscopic images of the GDD tubes and intraluminal threads were acquired using a multi-angle stereo microscope and digital camera system (VB-7010/VBG25, Keyence Co. Ltd., Osaka, Japan) under standardized magnification. Representative images utilized for morphometric analysis are displayed in Figure 2a–f. All measurements were performed using ImageJ, version 1.54g software (National Institutes of Health, Bethesda, MD, USA) based on calibrated microscopic images.

Although manufacturer-reported dimensions were recorded for device description, formula-derived pressure calculations were based on specimen-specific geometric measurements obtained from ImageJ analysis. For each specimen, the following parameters were measured from microscopic images: tube length (*L*), as well as the horizontal (*H*) and vertical (*V*) diameters of both the tube lumen and intraluminal thread. As the lumen and thread cross-sections were not found to be perfectly circular in all cases, the cross-sectional area (*A*) employed in the calculations was estimated using an elliptical approximation (Equation (1)) [16]:

(1) $$A=\left(\frac{\pi }{4}\right)\times H\times V,$$

In this equation, *H* and *V* are used to represent the horizontal and vertical diameters, respectively.

In the case of threaded conditions, the effective lumen area was calculated by subtracting the cross-sectional area of the intraluminal thread from that of the tube lumen. This method represented the remaining flow pathway available for fluid movement.

(2) $$\Delta P=\frac{8\pi \mu LQ}{A^{2}},$$

(3) $$\Delta P=\frac{8\mu LQ}{\pi {\left(\frac{A}{\pi }-\frac{A^{\prime }}{\pi }\right)}^{2}}\times \frac{1}{\ln\sqrt{\frac{A}{A^{\prime }}}},$$

Subsequently, formula-derived pressure values were calculated using established fluid dynamic models and the measured geometric parameters. In instances where an intraluminal thread was not present, the Hagen–Poiseuille equation was employed (Equation (2)) [16]. In threaded conditions, pressure was calculated using an annular flow model (Equation (3)), which describes laminar flow through the space between the tube wall and the intraluminal thread [16]. In these equations, Δ*P* denotes the pressure difference (Pa), *μ* signifies the dynamic viscosity of the fluid (Pa·s), *L* represents the length of the tube (m), *Q* denotes the flow rate (m^3^/s), *A* is the cross-sectional area of the tube lumen (m^2^), and *A*′ is the cross-sectional area of the intraluminal thread (m^2^). The resulting formula-derived pressure values were converted to mmHg for comparison with measured pressure values.

### 2.4. Statistical Analysis

All statistical analyses were performed using JMP Student Edition version 18 (SAS Institute Inc., Cary, NC, USA). Pressure–flow relationships under each experimental condition were analyzed and visualized graphically.

Linear mixed-effects regression models were used to evaluate pressure differences while accounting for repeated measurements across flow rates within each sample. For comparisons among thread conditions within each device, flow rate and thread condition were included as fixed effects, and SampleID was included as a random effect. Tukey-adjusted post hoc tests were applied for pairwise comparisons among thread conditions. For comparisons between measured and formula-derived pressure values, Type (Measured vs. Formula-Derived) and flow rate were included as fixed effects, with SampleID included as a random effect. Data are presented as mean ± standard deviation unless otherwise specified.

## 3. Results

**Figure 3:**
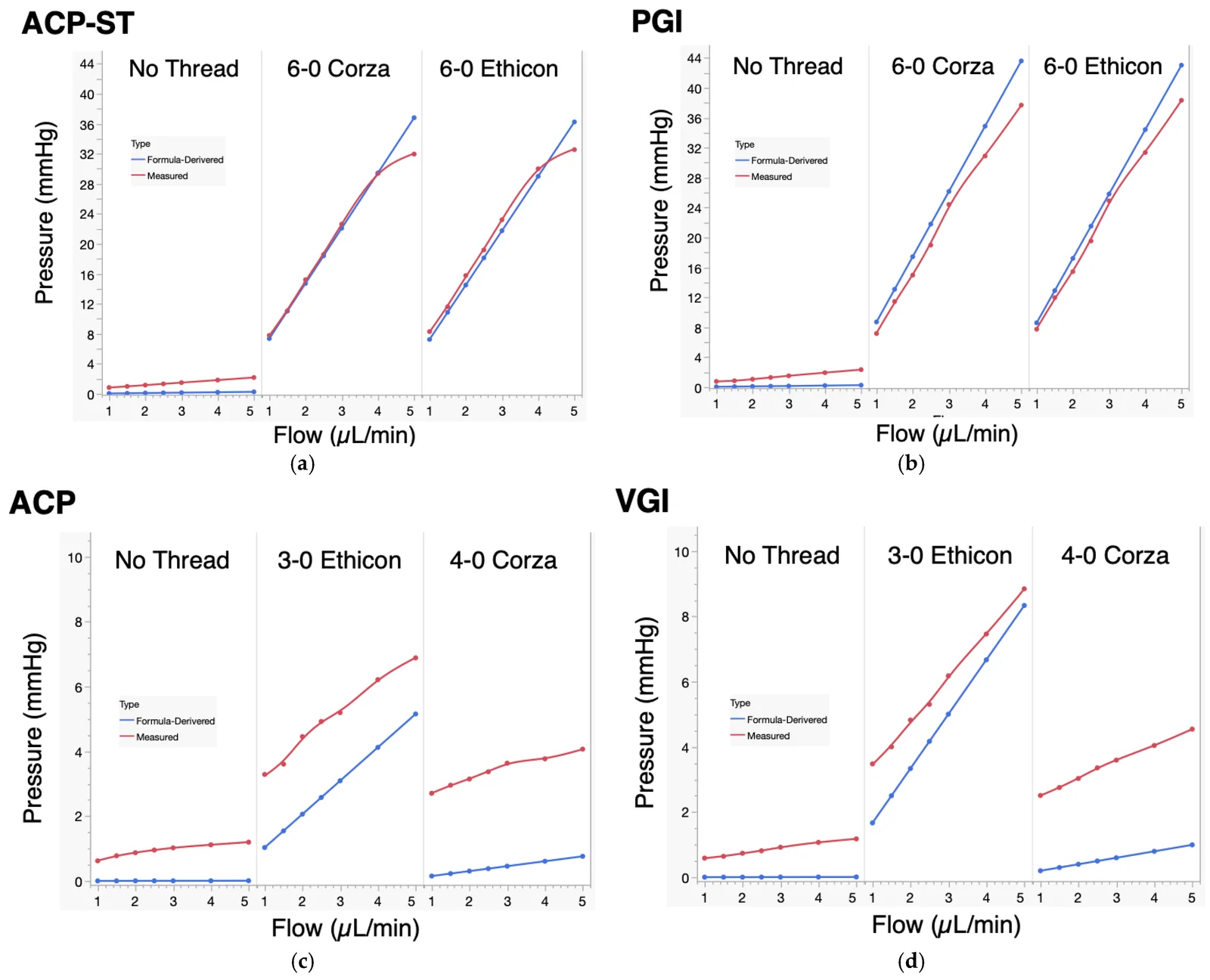
Pressure–flow profiles of four glaucoma drainage devices under different thread conditions. The mean pressure values (mmHg) are plotted against flow rates (1–5 µL/min) for (**a**) Ahmed ClearPath Small Tube, ACP-ST; (**b**) Paul Glaucoma Implant, PGI; (**c**) Ahmed ClearPath, ACP; and (**d**) Virna Glaucoma Implant, VGI. The red lines indicate measured pressure values, whereas the blue lines indicate formula-derived pressure values.

In Figure 3, the measured pressure (red lines) and the formula-derived pressure (blue lines) profiles of each device under the tested thread conditions are presented. For all devices and conditions, an increase in pressure was observed with increasing flow rate. Detailed pressure values at each flow rate are provided in Supplementary Table S1.

As demonstrated in the measured pressure profiles, intraluminal thread insertion resulted in increased pressure relative to the no-thread condition. In ACP-ST (Figure 3a) and PGI (Figure 3b), the 6-0 PP thread conditions (middle and right panels) exhibited higher pressure values in comparison to the no-thread condition (left panels). However, the two 6-0 PP thread conditions demonstrated analogous profiles. In ACP (Figure 3c) and VGI (Figure 3d), both threaded conditions increased pressure relative to the no-thread condition, with higher pressure values observed in the 3-0 PP Ethicon condition (middle panels) than in the 4-0 PP Corza condition (right panels). The formula-derived pressure profiles exhibited patterns similar to those of the measured pressure profiles. In ACP-ST and PGI, the insertion of 6-0 PP threads resulted in an increase in pressure relative to the no-thread condition, with minimal differences observed between Corza and Ethicon threads. In ACP and VGI, formula-derived pressure values were also higher in the 3-0 PP Ethicon condition than in the 4-0 PP Corza condition. A discrepancy was observed between the measured and formula-derived pressure values under a variety of conditions.

The results of the paired comparisons of the measured pressures among the various thread conditions are summarized in Table 1. In ACP-ST, both 6-0 PP thread conditions exhibited significantly higher pressure compared to the no-thread condition (ACP-ST [−] vs. ACP-ST [+6C], −18.11 mmHg, *p* < 0.001; ACP-ST [−] vs. ACP-ST [+6E], −18.68 mmHg, *p* < 0.001). Statistically significant differences were not observed between the two thread manufacturers (*p* = 0.85). In a similar manner, within the PGI framework, both 6-0 PP thread conditions yielded substantially elevated pressures in comparison to the no-thread condition (PGI [−] vs. PGI [+6C], −19.40 mmHg, *p* < 0.001; PGI [−] vs. PGI [+6E], −19.92 mmHg, *p* < 0.001). Conversely, no statistically significant difference was detected between Corza and Ethicon threads (*p* = 0.91). In ACP, all pairwise comparisons were statistically significant. The pressure levels were found to be considerably higher in the 3-0 PP Ethicon condition compared to the 4-0 PP Corza condition (ACP [+4C] vs. ACP [+3E], 1.56 mmHg, *p* < 0.001). A similar pattern was observed in VGI, in which all pairwise comparisons were significant (*p* < 0.001).

The mean differences between the measured and formula-derived pressure values are summarized in Table 2. In ACP-ST, the no-thread condition exhibited a substantial discrepancy between the measured and formula-derived pressure values (1.26 mmHg, *p* < 0.001), while no significant differences were observed under threaded conditions (6-0 PP Corza, −0.46 mmHg, *p* = 0.30; 6-0 PP Ethicon, 0.41 mmHg, *p* = 0.26). In contrast, PGI exhibited substantial discrepancies between measured and formula-derived values under all conditions, including the no-thread condition (1.25 mmHg, *p* < 0.001) and both threaded conditions (6-0 PP Corza, −2.87 mmHg, *p* < 0.001; 6-0 PP Ethicon, −2.03 mmHg, *p* < 0.001). ACP and VGI also exhibited substantial discrepancies between the measured and formula-derived pressure values under all conditions. In ACP, the mean differences were 0.93 mmHg in the no-thread condition, 2.97 mmHg with 4-0 PP Corza, and 2.14 mmHg with 3-0 PP Ethicon (*p* < 0.001). In VGI, the corresponding values were 0.84 mmHg, 2.86 mmHg, and 1.20 mmHg, respectively, with the 3-0 PP Ethicon condition showing a smaller but still significant deviation (*p* = 0.005).

## 4. Discussion

The present study quantified the hydraulic resistance among several recently introduced and emerging GDD designs, including ACP-ST, PGI, ACP, and VGI, highlighting the influence of tube geometry and thread insertion on pressure–flow behavior. Across all devices, a linear relationship between pressure and flow was consistently observed, supporting the assumption that tube resistance behaves predictably within the tested flow range [16].

In the absence of intraluminal threads, all devices demonstrated low resistance, as reflected by minimal pressure elevation across increasing flow rates. This finding is consistent with previous reports describing limited intrinsic resistance in unobstructed GDD tubes [16,26]. The introduction of intraluminal threads resulted in a marked increase in pressure across all devices, confirming their role as an effective method for modulating early postoperative outflow [16,27,28].

A key observation of this study is the different responses to thread insertion between devices with varying lumen geometries. In ACP-ST and PGI, which both incorporate a small-lumen design, the use of 6-0 PP threads resulted in substantial increases in pressure compared to the no-thread condition, with overlapping profiles between thread manufacturers. This finding suggests that in narrow lumens, resistance is predominantly influenced by the constrained flow pathway itself, such that minor discrepancies in thread dimensions exert a comparatively negligible impact on overall resistance.

In contrast, ACP and VGI exhibited more pronounced variation depending on thread size, with higher resistance observed in the 3-0 PP condition compared to the 4-0 PP thread. In these larger-lumen devices, the thread occupies a greater proportion of the effective lumen, leading to a more substantial reduction in the available flow area. From a fluid dynamics standpoint, this results in a disproportionate increase in resistance, as flow becomes more sensitive to local constriction effects rather than overall lumen size [16]. These findings indicate that hydraulic resistance in GDD tubes is not determined solely by either device geometry or intraluminal modification but by their interaction. In smaller-lumen devices, intrinsic resistance dominates, whereas in larger-lumen devices, resistance becomes increasingly dependent on thread characteristics.

From a clinical perspective, the similar resistance profiles observed between 6-0 Corza and 6-0 Ethicon threads suggest that manufacturer-related differences may have a limited impact on tube resistance when the same nominal USP thread size is used in small-lumen devices. Accordingly, the differences in resistance observed between the 3-0 Ethicon and 4-0 Corza threads in the ACP and VGI are likely attributable primarily to the difference in thread size rather than to manufacturer-related differences. However, tube resistance represents only one component of postoperative pressure regulation. The final postoperative IOP cannot be predicted from tube resistance alone because plate surface area, plate shape, bleb formation, tissue encapsulation, and individual wound-healing responses also contribute substantially to the final pressure outcome after GDD implantation [29,30]. Therefore, even when a larger intraluminal thread such as 3-0 PP is used to increase tube resistance, additional flow-restricting techniques, including external ligation, may still be necessary depending on the device and clinical situation [16,31].

From a fluid mechanics perspective, the observed linear pressure–flow relationships support the applicability of simplified models such as Hagen–Poiseuille flow under the experimental conditions. However, the correlation between measured and formula-derived pressure exhibited variability among devices and thread conditions. In ACP-ST, measured and formula-derived pressure values did not differ significantly under threaded conditions. Significant differences were observed in PGI, although the overall pressure–flow profiles of the two devices were visually similar. Similarly, ACP and VGI exhibited substantial deviations under all tested conditions, though the extent of these deviations differed based on device and thread configuration. These findings suggest that the geometry of tubes and threads alone does not fully explain the hydraulic behavior of all devices. While formula-derived calculations provide a useful approximation of resistance, device-specific factors may influence the resulting pressure–flow relationship. The observed discrepancy in the levels of agreement between ACP-ST and PGI, as well as between ACP and VGI, suggests the possibility of distinct intraluminal flow behavior in devices with apparently similar lumen concepts under experimental conditions.

The discrepancies between these measured values and the formula-derived values can be attributed to several factors. The formula-derived calculations were based on an idealized annular flow model that assumes a uniform geometry and a concentric flow channel between the tube wall and the intraluminal thread. However, microscopic observations indicated that the thread was not consistently centrally positioned within the tube lumen. In cross-sectional images, the threads appeared eccentric within the lumen in ACP-ST, ACP, and VGI (Figure 2a–c). Furthermore, longitudinal imaging of the PGI tube revealed that this eccentric positioning was present not only at the tube opening but also along the tube lumen (Figure 2e,f). Such asymmetric and non-uniform configurations may create uneven flow channels around the thread, resulting in flow behavior that deviates from the assumptions of an ideal concentric annular model. Consequently, the formula-derived values should be interpreted as theoretical approximations rather than exact representations of actual intraluminal flow behavior [16].

The observed discrepancies between devices highlight the necessity of experimental validation when assessing flow resistance in glaucoma drainage devices. While formula-derived calculations provide a useful mechanistic framework, actual hydraulic behavior may be influenced by factors that are not readily captured by simplified geometric models. Consequently, formula-derived estimates should be interpreted as approximations rather than exact representations of intraluminal flow resistance.

It is important to acknowledge the limitations of this study. First, measurements were conducted under controlled in vitro conditions using saline, which does not fully replicate the viscosity and biochemical properties of aqueous humor. Moreover, all measurements were performed at 25 °C rather than at the physiological temperature of 37 °C. Because saline viscosity decreases moderately with increasing temperature, the absolute resistance under physiological conditions may be lower than that measured in this study; however, this effect is unlikely to alter the relative comparisons because all devices were evaluated under identical temperature conditions. In addition, the flow-rate range of 1–5 µL/min was selected to evaluate the pressure–flow characteristics of each device over an IOP-relevant pressure range, rather than to reproduce physiological aqueous humor production. Therefore, particularly at the higher flow rates, the experimental conditions should be interpreted as pressure test conditions for characterizing device resistance rather than as physiological flow conditions. Second, the intraluminal threads were inserted without fixation, which may have introduced variability in thread positioning. Third, the thread conditions evaluated were selected to reflect clinically relevant configurations currently in use with each device. Consequently, the findings may not be directly applicable to alternative thread sizes or configurations that were not examined in this study. Fourth, the sample size per device was limited, although independent devices were used to ensure measurement independence. Finally, this study focused on short-term flow behavior and did not account for postoperative factors such as bleb formation or tissue response. It is recommended that subsequent studies evaluate these findings under in vivo or clinically relevant conditions to ascertain how tube resistance interacts with biological factors in regulating postoperative IOP. Despite the inherent limitations of this study, it provides a direct comparative evaluation of tube resistance across multiple GDDs under standardized conditions. By isolating the contributions of tube geometry and thread insertion, the results provide further insight into how device design influences early postoperative flow dynamics.

## 5. Conclusions

In conclusion, intraluminal PP thread insertion significantly increased tube resistance in all GDDs evaluated, although the magnitude of resistance depended on the interaction between thread size and tube geometry. In small-lumen devices, manufacturer-related differences between 6-0 PP threads had minimal influence on hydraulic resistance, whereas in larger-lumen devices, thread size produced measurable differences in pressure–flow behavior. Agreement between measured and formula-derived pressure varied among devices, indicating that theoretical models based solely on tube and thread geometry may not fully capture actual intraluminal flow characteristics. These findings improve the understanding of the hydraulic behavior of contemporary GDDs and may assist surgeons in selecting appropriate intraluminal thread strategies for early postoperative flow modulation.

## Figures and Tables

**Table 1:** Pairwise comparisons of measured pressure among thread conditions within each device.

Glaucoma Drainage Device	Mean Difference (95% CI)	*p* Value
**Ahmed ClearPath Small Tube**		
ACP-ST (−) vs. ACP-ST (+6C)	−18.11 (−20.67 to −15.55)	<0.001 **
ACP-ST (−) vs. ACP-ST (+6E)	−18.68 (−21.24 to −16.12)	<0.001 **
ACP-ST (+6C) vs. ACP-ST (+6E)	−0.57 (−3.13 to 1.99)	0.85
**Paul Glaucoma Implant**		
PGI (−) vs. PGI (+6C)	−19.40 (−22.46 to −16.33)	<0.001 **
PGI (−) vs. PGI(+6E)	−19.92 (−22.99 to −16.86)	<0.001 **
PGI (+6C) vs. PGI (+6E)	−0.53 (−3.60 to 2.54)	0.91
**Ahmed ClearPath**		
ACP (−) vs. ACP (+4C)	−2.44 (−2.80 to −2.10)	<0.001 **
ACP (−) vs. ACP (+3E)	−4.00 (−4.36 to −3.64)	<0.001 **
ACP (+4C) vs. ACP (+3E)	1.56 (1.20 to 1.92)	<0.001 **
**Virna Glaucoma Implant**		
VGI (−) vs. VGI (+4C)	−2.55 (−3.00 to −2.11)	<0.001 **
VGI (−) vs. VGI (+3E)	−4.88 (−5.32 to −4.43)	<0.001 **
VGI (+4C) vs. VGI (+3E)	−2.32 (1.88 to 2.77)	<0.001 **

Data are presented as mean differences between conditions (mmHg) with 95% confidence intervals. Pairwise comparisons are performed using linear mixed-effects regression with Tukey adjustment. E and C denote Ethicon and Corza polypropylene threads, respectively; numerical designations indicate thread size and (−) denotes no intraluminal thread. ** *p* < 0.01.

**Table 2:** Differences between measured and formula-derived pressure values within each device and thread condition.

Glaucoma Drainage Device	Condition	Mean Difference (95% CI)	*p* Value
**Ahmed ClearPath Small Tube**	No Thread	1.26 (1.15 to 1.36)	<0.001 **
	Thread 6-0 Corza	−0.46 (−1.35 to 0.42)	0.30
	Thread 6-0 Ethicon	0.41 (−0.31 to 1.14)	0.26
**Paul Glaucoma Implant**	No Thread	1.25 (1.12 to 1.39)	<0.001 **
	Thread 6-0 Corza	−2.87 (−3.88 to 1.86)	<0.001 **
	Thread 6-0 Ethicon	−2.03 (−3.03 to −1.03)	<0.001 **
**Ahmed ClearPath**	No Thread	0.93 (0.87 to 1.00)	<0.001 **
	Thread 4-0 Corza	2.97 (2.87 to 3.06)	<0.001 **
	Thread 3-0 Ethicon	2.14 (1.57 to 2.70)	<0.001 **
**Virna Glaucoma Implant**	No Thread	0.84 (0.78 to 0.91)	<0.001 **
	Thread 4-0 Corza	2.86 (2.74 to 2.99)	<0.001 **
	Thread 3-0 Ethicon	1.20 (0.39 to 2.01)	0.005 **

Mean differences are presented as measured pressure minus formula-derived pressure (mmHg), with 95% confidence intervals. *p* values were obtained using linear mixed-effects regression with type (measured vs. formula-derived) and flow rate as fixed effects. ** *p* < 0.01.

## Data Availability

The raw data supporting the conclusions of this article are available from the corresponding author upon reasonable request.

## Supplementary Materials

The following supporting information can be downloaded at https://www.mdpi.com/article/10.3390/bioengineering13080923/s1, Supplementary Table S1. Pressure values at various flow rates in ACP-ST, PGI, ACP, and VGI.

## Abbreviations

The following abbreviations are used in this manuscript:

ACP	Ahmed ClearPath
ACP-ST	Ahmed ClearPath Small Tube
GDD	Glaucoma Drainage Device
IOP	Intraocular Pressure
PGI	Paul Glaucoma Implant
PP	Polypropylene
USP	United States Pharmacopeia
VGI	Virna Glaucoma Implant
