# Supplementary material for: Comparative In Vitro Evaluation of Tube Resistance in Glaucoma Drainage Devices with Intraluminal Polypropylene Threads
Source: Bioengineering (Basel). 2026 Aug 14;13(8):923. doi: 10.3390/bioengineering13080923 (PMC13510013; doi:10.3390/bioengineering13080923)
Supplement: Supplementary file 1 [file bioengineering-13-00923-s001.zip › bioengineering-4443315-supplementary.pdf]

Supplementary Table S1. Pressure Values at Various Flow Rates in ACP-ST, PGI, ACP, and VGI

**Measured Pressure Values**												
**Flow**	**ACP-ST**			**PGI**			**ACP**			**VGI**		
**(µL/min)**	**(-)**	**(+6E)**	**(+6C)**	**(-)**	**(+6E)**	**(+6C)**	**(-)**	**(+4C)**	**(+3E)**	**(-)**	**(+4C)**	**(+3E)**
1.0	0.84 ± 0.06	8.30 ± 0.10	7.79 ± 0.06	0.78 ± 0.11	7.75 ± 0.32	7.17 ± 0.29	0.62 ± 0.12	2.70 ± 0.35	3.29 ± 0.10	0.59 ± 0.07	2.51 ± 0.15	3.48 ± 0.05
1.5	0.99 ± 0.06	11.61 ± 0.14	11.04 ± 0.07	0.85 ± 0.12	11.97 ± 0.70	11.45 ± 0.73	0.78 ± 0.06	2.96 ± 0.15	3.61 ± 0.33	0.64 ± 0.08	2.75 ± 0.15	4.00 ± 0.04
2.0	1.16 ± 0.05	15.77 ± 0.10	15.21 ± 0.11	1.07 ± 0.10	15.44 ± 0.70	14.98 ± 0.75	0.87 ± 0.08	3.15 ± 0.13	4.46 ± 0.81	0.74 ± 0.09	3.03 ± 0.22	4.82 ± 0.07
2.5	1.33 ± 0.07	19.18 ± 0.09	18.58 ± 0.07	1.30 ± 0.13	19.54 ± 2.25	18.99 ± 2.24	0.95 ± 0.10	3.37 ± 0.14	4.92 ± 0.82	0.81 ± 0.11	3.36 ± 0.22	5.30 ± 0.05
3.0	1.50 ± 0.09	23.20 ± 0.09	22.62 ± 0.08	1.52 ± 0.14	24.90 ± 1.18	24.42 ± 1.16	1.02 ± 0.07	3.63 ± 0.09	5.20 ± 0.66	0.92 ± 0.14	3.59 ± 0.20	6.18 ± 0.10
4.0	1.84 ± 0.11	30.00 ± 0.17	29.39 ± 0.12	1.94 ± 0.07	31.36 ± 1.07	30.89 ± 1.06	1.12 ± 0.10	3.77 ± 0.13	6.21 ± 0.42	1.07 ± 0.11	4.04 ± 0.21	7.46 ± 0.04
5.0	2.18 ± 0.12	32.55 ± 0.25	31.96 ± 0.17	2.34 ± 0.05	38.33 ± 1.83	37.68 ± 1.80	1.20 ± 0.15	4.07 ± 0.25	6.89 ± 0.60	1.18 ± 0.06	4.54 ± 0.16	8.84 ± 0.06
	vs. (-)	p < 0.001**	p < 0.001**	vs. (-)	p < 0.001**	p < 0.001**	vs. (-)	p < 0.001**	p < 0.001**	vs. (-)	p < 0.001**	p < 0.001**
	vs. (+6E)		p = 0.85	vs. (+6E)		p = 0.91	vs. (+4C)		p < 0.001**	vs. (+4C)		p < 0.001**
**Formula-Derived Pressure Values**												
**Flow**	**ACP-ST**			**PGI**			**ACP**			**VGI**		
**(µL/min)**	**(-)**	**(+6E)**	**(+6C)**	**(-)**	**(+6E)**	**(+6C)**	**(-)**	**(+4C)**	**(+3E)**	**(-)**	**(+4C)**	**(+3E)**
1.0	0.05 ± 0.00	7.25 ± 0.51	7.36 ± 0.77	0.05 ± 0.00	8.60 ± 0.87	8.72 ± 0.84	0.00 ± 0.00	0.15 ± 0.04	1.03 ± 0.57	0.00 ± 0.00	0.20 ± 0.04	1.67 ± 0.99
1.5	0.08 ± 0.00	10.87 ± 0.76	11.04 ± 1.16	0.08 ± 0.00	12.91 ± 1.31	13.08 ± 1.27	0.00 ± 0.00	0.23 ± 0.06	1.55 ± 0.86	0.00 ± 0.00	0.30 ± 0.05	2.50 ± 1.49
2.0	0.11 ± 0.00	14.50 ± 1.02	14.72 ± 1.54	0.11 ± 0.00	17.21 ± 1.75	17.44 ± 1.69	0.00 ± 0.00	0.30 ± 0.07	2.06 ± 1.14	0.00 ± 0.00	0.40 ± 0.07	3.34 ± 1.99
2.5	0.13 ± 0.00	18.12 ± 1.27	18.40 ± 1.93	0.14 ± 0.00	21.51 ± 2.18	21.80 ± 2.11	0.00 ± 0.00	0.38 ± 0.09	2.58 ± 1.43	0.00 ± 0.00	0.50 ± 0.09	4.17 ± 2.49
3.0	0.16 ± 0.01	21.74 ± 1.53	22.08 ± 2.31	0.16 ± 0.00	25.81 ± 2.62	26.16 ± 2.53	0.00 ± 0.00	0.46 ± 0.11	3.09 ± 1.71	0.00 ± 0.00	0.60 ± 0.11	5.00 ± 2.98
4.0	0.21 ± 0.01	28.99 ± 2.03	29.44 ± 3.09	0.22 ± 0.00	34.42 ± 3.49	34.87 ± 3.38	0.01 ± 0.00	0.61 ± 0.15	4.12 ± 2.29	0.01 ± 0.00	0.80 ± 0.14	6.67 ± 3.98
5.0	0.27 ± 0.01	36.24 ± 2.54	36.80 ± 3.86	0.27 ± 0.00	43.02 ± 4.36	43.59 ± 4.22	0.01 ± 0.00	0.76 ± 0.18	5.15 ± 2.86	0.01 ± 0.00	0.99 ± 0.18	8.34 ± 4.97
	vs. (-)	p < 0.001**	p < 0.001**	vs. (-)	p < 0.001**	p < 0.001**	vs. (-)	p = 0.27	p < 0.001**	vs. (-)	p = 0.46	p < 0.001**
	vs. (+6E)		p = 0.97	vs. (+6E)		p = 0.98	vs. (+4C)		p < 0.001**	vs. (+4C)		p < 0.001**
**Difference Pressure Values**												
**Flow**	**ACP-ST**			**PGI**			**ACP**			**VGI**		
**(µL/min)**	**(-)**	**(+6E)**	**(+6C)**	**(-)**	**(+6E)**	**(+6C)**	**(-)**	**(+4C)**	**(+3E)**	**(-)**	**(+4C)**	**(+3E)**
1.0	0.79 ± 0.06	1.05 ± -0.41	0.43 ± -0.71	0.73 ± 0.11	-0.85 ± -0.55	-1.55 ± -0.55	0.62 ± 0.12	2.55 ± 0.31	2.26 ± -0.47	0.59 ± 0.07	2.31 ± 0.11	1.81 ± -0.94
1.5	0.91 ± 0.06	0.74 ± -0.62	0.00 ± -1.09	0.77 ± 0.12	-0.94 ± -0.61	-1.63 ± -0.54	0.78 ± 0.06	2.73 ± 0.09	2.06 ± -0.53	0.64 ± 0.08	2.45 ± 0.10	1.50 ± -1.45
2.0	1.05 ± 0.05	1.27 ± -0.92	0.49 ± -1.43	0.96 ± 0.10	-1.77 ± -1.05	-2.46 ± -0.94	0.87 ± 0.08	2.85 ± 0.06	2.4 ± -0.33	0.74 ± 0.09	2.63 ± 0.15	1.48 ± -1.92
2.5	1.2 ± 0.07	1.06 ± -1.18	0.18 ± -1.86	1.16 ± 0.13	-1.97 ± -0.07	-2.81 ± 0.13	0.95 ± 0.10	2.99 ± 0.05	2.34 ± -0.61	0.81 ± 0.11	2.86 ± 0.13	1.13 ± -2.44
3.0	1.34 ± 0.08	1.46 ± -1.44	0.54 ± -2.23	1.36 ± 0.14	-0.91 ± -1.44	-1.74 ± -1.37	1.02 ± 0.07	3.17 ± -0.02	2.11 ± -1.05	0.92 ± 0.14	2.99 ± 0.09	1.18 ± -2.88
4.0	1.63 ± 0.10	1.01 ± -1.86	-0.05 ± -2.97	1.72 ± 0.07	-3.06 ± -2.42	-3.98 ± -2.32	1.11 ± 0.10	3.16 ± -0.02	2.09 ± -1.87	1.06 ± 0.11	3.24 ± 0.07	0.79 ± -3.94
5.0	1.91 ± 0.11	-3.69 ± -2.29	-4.84 ± -3.69	2.07 ± 0.05	-4.69 ± -2.53	-5.91 ± -2.42	1.19 ± 0.15	3.31 ± 0.07	1.74 ± -2.26	1.17 ± 0.06	3.55 ± -0.02	0.50 ± -4.91
Measured vs. Formula-derived	p < 0.001**	p = 0.30	p = 0.26	p < 0.001**	p < 0.001**	p < 0.001**	p < 0.001**	p < 0.001**	p < 0.001**	p < 0.001**	p < 0.001**	p = 0.005**

Values are presented as mean ± standard deviation (mmHg). Flow rate is expressed in µL/min. Difference pressure values are calculated as measured pressure minus formula-derived pressure. (A) Ahmed ClearPath Small Tube, ACP-ST; (B) Paul Glaucoma Implant, PGI; (C) Ahmed ClearPath, ACP; and (D) Virna Glaucoma Implant, VGI. E and C denote Ethicon and Corza polypropylene threads, respectively; numerical designations indicate thread size, and (−) denotes no intraluminal thread. P values are obtained using linear mixed-effects regression for measured versus formula-derived pressure comparisons and Tukey-adjusted post hoc tests for comparisons among thread conditions.

** P < 0.01
